# Genes for laminarin degradation are dispersed in the genomes of particle-associated *Maribacter* species

**DOI:** 10.3389/fmicb.2024.1393588

**Published:** 2024-08-12

**Authors:** Saskia Kalenborn, Daniela Zühlke, Greta Reintjes, Katharina Riedel, Rudolf I. Amann, Jens Harder

**Affiliations:** ^1^Department of Molecular Ecology, Max Planck Institute for Marine Microbiology, Bremen, Germany; ^2^Department for Microbial Physiology and Molecular Biology, University of Greifswald, Greifswald, Germany; ^3^Microbial Carbohydrate Interaction Group, Department of Biology and Chemistry, University of Bremen, Bremen, Germany

**Keywords:** polysaccharide utilization locus, GH16, laminarinase, *Flavobacteriia*, marine

## Abstract

Laminarin is a cytosolic storage polysaccharide of phytoplankton and macroalgae and accounts for over 10% of the world’s annually fixed carbon dioxide. Algal disruption, for example, by viral lysis releases laminarin. The soluble sugar is rapidly utilized by free-living planktonic bacteria, in which sugar transporters and the degrading enzymes are frequently encoded in polysaccharide utilization loci. The annotation of flavobacterial genomes failed to identify canonical laminarin utilization loci in several particle-associated bacteria, in particular in strains of *Maribacter*. In this study, we report *in vivo* utilization of laminarin by *Maribacter forsetii* accompanied by additional cell growth and proliferation. Laminarin utilization coincided with the induction of an extracellular endo-laminarinase, SusC/D outer membrane oligosaccharide transporters, and a periplasmic glycosyl hydrolase family 3 protein. An ABC transport system and sugar kinases were expressed. Endo-laminarinase activity was also observed in *Maribacter* sp. MAR_2009_72, *Maribacter* sp. Hel_I_7, and *Maribacter dokdonensis* MAR_2009_60. *Maribacter dokdonensis* MAR_2009_71 lacked the large endo-laminarinase gene in the genome and had no endo-laminarinase activity. In all genomes, genes of induced proteins were scattered across the genome rather than clustered in a laminarin utilization locus. These observations revealed that the *Maribacter* strains investigated in this study participate in laminarin utilization, but in contrast to many free-living bacteria, there is no co-localization of genes encoding the enzymatic machinery for laminarin utilization.

## Introduction

Laminarin is a major carbon storage polysaccharide in algae. Annual net biosynthesis rates have been estimated to be in the range of 6–18 Gt carbon, one–eighth of the annual biomass production on earth (Alderkamp et al., 2007; Becker et al., 2020). The water-soluble compound is a linear glucose polymer with decorations. Approximately 25 glucose molecules are linked by β-(1,3)-glycosidic bonds and modified in algal species with β-(1,6)-linked glucose side chains (Nelson and Lewis, 1974; Alderkamp et al., 2007; Rioux et al., 2007; Kadam et al., 2015; Becker et al., 2020). It becomes available as an important carbon source for microorganisms when algae are lysed by zooplankton predation or viral infection. Then, as part of the dissolved organic carbon fraction (DOC), it sustains bacterioplankton blooms following algal blooms in regions with a dynamic annual climate, i.e. temperate and polar regions (Teeling et al., 2012, 2016; Sidhu et al., 2023). DOC favors growth of free-living bacteria, and the abundance of particle-attached bacteria remains low (Heins et al., 2021b). In bacteria of the phylum *Bacteroidota*, polysaccharide utilization genes are often colocalized in genomic loci or islands, termed polysaccharide utilization loci (PUL) (Grondin et al., 2017).

The starch utilization system (Sus) was the first described PUL (Shipman et al., 2000). Efficient polysaccharide degradation pathways include extracellular hydrolysis, preferentially at the cell surface including forwarding of lysis products to a transporter. The oligosaccharides are actively translocated into the periplasm where they are hydrolyzed and the monomers are transported into the cytoplasm. The outer membrane transport system SusC/D receives its energy from a proton gradient via an ExbBD-TonB system in the cytoplasmic membrane and a periplasmic domain to open the transport channel on the periplasmic side (Noinaj et al., 2010). The depolymerization of marine polysaccharides is catalyzed by glycoside hydrolases, glycosyltransferases, polysaccharide lyases, and carbohydrate esterases which digest the polysaccharides with a high specificity, often assisted by carbohydrate-binding modules in multidomain proteins. These protein groups are summarized as carbohydrate-active enzymes (CAZymes) (Bäumgen et al., 2021; Drula et al., 2022). Anionic polymers require additional enzymes including sulfatases for utilizing ulvan, carrageenan, porphyran, and fucose-containing polysscharides. For laminarin, up to three PULs have been found in a single genome in free-living bacteria, in example in *Formosa* clade B (Unfried et al., 2018). An *in-silico* study on 53 flavobacterial genomes identified PULs for laminarin utilization in free-living flavobacterial strains, but not in 10 out of 12 strains in a clade of genera of particle-associated strains, mainly isolated from a 20 μm particle net catch (Kappelmann et al., 2019).

Particle-associated bacteria include chemotactic motile free-living bacteria and particle-attached bacteria (Heins et al., 2021a,b). From the genera identified by size filtration and diatom settlement experiments as strongly particle-associated, we selected *Maribacter* to study laminarin degradation. *Maribacter* was first described in 2004 as a new genus in the family *Flavobacteriaceae* (Nedashkovskaya et al., 2004). In nine samples taken over a spring algal bloom, 56 *Maribacter* strains were among 3,572 isolated strains isolated from bulk seawater (Alejandre-Colomo et al., 2020). In large comparative studies, *Maribacter* strains were predominantly isolated from particle fractions (>3 μm filter retentate) and rarely from bulk seawater which was not size-fractionated and contained the natural population of particles (Heins and Harder, 2023; Lu et al., 2023). Molecular community analyses showed that *Maribacter* are rare in free-living bacterial populations as defined by bacterioplankton fraction of 0.2–3 μm size in sequential filtration (Sidhu et al., 2023, and references therein). Abundances of up to 4% were detected in surface populations of oxic sandy sediment (Probandt et al., 2018; Miksch et al., 2021). Higher relative abundances were reported for phycosphere populations on micro- and macroalgae (Heins et al., 2021b; Lu et al., 2023; Wang et al., 2024). In addition, being a dominant epiphyte on many algae, *Maribacter* synthesize with thallusin a controlling factor of *Ulva* morphogenesis. This demonstrates the interplay of particle-associated bacteria with algae in the phycosphere (Alsufyani et al., 2020).

Currently, 32 validly described species and more than 180 genomes have been published. In this study, two strains from seawater—*Maribacter forsetii* DSM 18668^T^ (Barbeyron et al., 2008) and *Maribacter* sp. Hel_I_7 from the long-term ecological research station Kabeltonne near Helgoland—were investigated together with three strains from phytoplankton catches in the Wadden Sea near Sylt—*Maribacter* sp. MAR_2009_72, *Maribacter dokdonensis* MAR_2009_60, and *Maribacter dokdonensis* MAR_2009_71 (Hahnke and Harder, 2013). For all these strains representing four species (Supplementary Table S1), the genomes have been published, which opened the opportunity to use proteomics for the identification and location of genes and proteins involved in laminarin degradation. We detected that genes for laminarin degradation are dispersed in the genomes of four particle-associated *Maribacter* species.

## Materials and methods

### Growth experiments

*Maribacter forsetii* DSM 18668^T^ (Barbeyron et al., 2008), *Maribacter* sp. Hel_I_7 (DSM 29657), *Maribacter* sp. MAR_2009_72 (DSM 29384), *Maribacter dokdonensis* MAR_2009_60 (DSM 29383), and *Maribacter dokdonensis* MAR_2009_71 (DSM 29385) (Supplementary Table S1) were reactivated from glycerol stocks maintained in the laboratory since the isolation of the strains (Hahnke et al., 2013, 2015). All strains were grown in a liquid HaHa_100V medium with 0.3 g/L casamino acids as sole carbon source, limiting the growth to an optical density (OD) at 600 nm below 0.2 (Hahnke et al., 2015). The addition of 2 g/L of a carbohydrate source enabled growth beyond OD of 0.3. Laminarin from *Laminaria digitata*, glucose, and galactose (only for *Maribacter* sp. MAR_2009_72) (Sigma-Aldrich/Merck KGaA, Darmstadt, Germany) were used in growth experiments. In 250 mL photometer-sidearm Erlenmeyer flasks, 50 mL cultures were inoculated with 0.4% v/v of a pre-grown culture in the same medium and incubated with 110 rpm at room temperature. In addition to three cultures for proteomic analyses, a fourth culture was used to monitor bacterial growth by measuring optical and cell density. Cells were harvested at an OD of 0.15 for casamino acid cultures serving as control cultures and at an OD of 0.25 for sugar-containing cultures. Cells were pelleted by centrifugation in 50 mL tubes with 3,080 × *g* for 30 min at 4°C. Pellets were resuspended in 1 mL medium and centrifuged in 1.5 mL tubes at 15,870 × *g* for 15 min at 4°C. The wet biomass was weighed and stored at −20°C.

To determine cell density, the cells were filtered onto a 47 mm polycarbonate filter (0.2 μm pore size, Millipore, Darmstadt, Germany), applying a vacuum of 200 mbar. For microscopic cell counting, filter pieces were stained with DAPI (4,6-diamidino-2-phenylindole, Sigma-Aldrich, Steinheim, Germany) at a final concentration of 1 μg/mL. Subsequently, the filters were embedded in an antifading mounting solution of CitiFluor and Vectashield (3/1v/v) (CitiFluor Ltd., London, United Kingdom; Vector Laboratories, Inc., Burlingame, CA, United States). The cell abundance was determined by counting the number of DAPI signals per counting grid with a defined area. A minimum of 30 grids were counted per filter piece (>280 cells), and the total cell abundance was calculated by scaling the grid to the total filter area and divided by the sample volume.

### Saccharide uptake visualization

Uptake studies with fluorescently labeled laminarin were performed with *Maribacter* strains, *Christiangramia* (formerly known as *Gramella*) *forsetii* DSM17595 as positive control and *Escherichia coli* DSM498 as negative control. *Flavobacteriia* were grown on carbon-limited HaHa_100V medium with 0.3 g/L casamino acids as sole carbon source (CAA medium) or including 20 mg/L laminarin to obtain induced cells. *E. coli* was grown in one-tenth of the marine medium (10% CAA medium in water) containing 0.5 g/L tryptone and 0.25 g/L yeast extract. Uptake experiments with 40 μM labeled laminarin were inoculated with OD (600 nm) = 0.01. Non-induced and induced cells were also incubated without the labeled laminarin to determine cellular autofluorescence. Then, 1 mL samples were taken after 1 min, 1, 2, 4, and 24 h. After 1 h of fixation with 1% v/v formaldehyde, samples were diluted with 5 mL medium and cells were filtered onto 0.2 μm filter (diameter 47 mm) and stained with DAPI (1 ng/μl) for automatic microscopy (Reintjes et al., 2017).

Automated imaging of cells used a Zeiss AxioImager.Z2 microscope stand (Carl Zeiss, Oberkochen, Germany) as described in Bennke et al. (2016). A 63 × magnification and 1.4 numerical aperture oil emersion plan apochromatic objective (Carl Zeiss) was used with fixed exposure times of 50, 100, and 150 ms. Images were not optimized for Min/Max display normalization but acquired in linear range 0–255 (based on an 8-bit gray scale) to facilitate cross-comparison of signal intensities on images. Cell detection was performed using the ACMEtool software for automated cell enumeration (Bennke et al., 2016).^1^ Cells were identified by their DAPI signal, and a signal at the same location on the filter in the laminarin images was considered as labeled cell. Positive cells had a minimum overlap of 30% area and a minimum area of 20 pixels (0.3 μm^2^) (Reintjes et al., 2017).

### Protein preparation and mass spectrometry

Proteins were extracted from cells using a bead-beating method (Schultz et al., 2020). A pellet of 20–200 mg wet weight was disintegrated with 0.25 mL glass beads (0.1 mm) in 500 μL lysis buffer. Protein was quantified using the Roti-Nanoquant assay (Carl Roth, Karlsruhe, Germany). For protein separation on denaturing polyacrylamide gels (SDS-PAGE), 50 μg of protein was mixed with 10 μL of 4x SDS buffer (20% glycerol, 100 mM Tris/HCl, 10% (w/v) SDS, 5% β-mercaptoethanol, 0.8% bromophenol blue, pH 6.8) and loaded on Tris-glycine-extended (TGX) precast 4–20% gels (Biorad, Neuried, Germany). Electrophoresis was performed for 8 min at 150 V. The gel was fixed in 10% v/v acetic acid and 40% v/v ethanol for 30 min and stained with Brilliant Blue G250 Coomassie, and the protein band was excised. The proteins were dissected in one gel piece, and the pieces were washed with 50 mM ammonium bicarbonate in 30% v/v acetonitrile. Evaporation in a SpeedVac (Eppendorf, Hamburg, Germany) yielded dry gel pieces which were reswollen with 2 ng/μl trypsin (sequencing grade trypsin, Promega, United States). After 15-min incubation at room temperature, the excess liquid was removed. Samples were digested overnight at 37°C. The gel pieces were then covered with MS-grade water, and peptides were eluted with ultrasonication. The peptides were desalted using Pierce™ C18 Spin Tips (Thermo Fisher, Schwerte, Germany) according to the manufacturer’s guidelines. The eluates were dried in the SpeedVac and then stored at −20°C. For the MS analysis, the samples were thawed and taken up in 10 μL of Buffer A (99.9% acetonitrile +0.1% acetic acid).

Tryptic peptides of *Maribacter forsetii* and *Maribacter* sp. MAR_2009_72 samples were analyzed on an EASY-nLC 1,200 coupled to a Q Exactive HF mass spectrometer (Thermo Fisher Scientific, Waltham, United States). Peptides were loaded onto a self-packed analytical column (3 μm C18 particles, Dr. Maisch GmbH, Ammerbuch, Germany) using buffer A (0.1% acetic acid) with a flow rate of 2 μL/min within 5 min and separated using an 85 min binary gradient from 4 to 50% buffer B (0.1% acetic acid in acetonitrile) and a flow rate of 300 nL/min. Samples were measured in parallel mode; survey scans in the Orbitrap were recorded with a resolution of 60,000 with m/z of 333–1,650. The 15 most intense peaks per scan were selected for fragmentation. Precursor ions were dynamically excluded from fragmentation for 30 s. Single-charged ions and ions with unknown charge states were rejected. Internal lock mass calibration was applied (lock mass 445.12003 Da).

LC–MS/MS analyses of *Maribacter* sp. Hel_I_7, *Maribacter dokdonensis* MAR_2009_60, and *Maribacter dokdonensis* MAR_2009_71 samples were carried out on an EASY-nLC II coupled to a LTQ XL Orbitrap mass spectrometer (Thermo Fisher Scientific, Waltham, United States). Peptides were loaded onto a self-packed analytical column (3 μm C18 particles, Dr. Maisch GmbH, Ammerbuch, Germany) using buffer A (0.1% acetic acid) with a flow rate of 500 nL/min and separated using a 56 min binary gradient from 5 to 40% buffer B (0.1% acetic acid in acetonitrile) and a flow rate of 300 nL/min. Samples were measured in parallel mode; survey scans in the Orbitrap were recorded with a resolution of 30,000 with m/z of 300–2,000. The 5 most intense peaks per scan were selected for fragmentation. Precursor ions were dynamically excluded from fragmentation for 30 s. Single-charged ions and ions with unknown charge states were rejected. Internal lock mass calibration was applied (lock mass 445.12003 Da).

The mass spectrometry files were analyzed in MaxQuant version 2.2.0.0 in the standard settings—see Supplement information for details—against the strain-specific protein database downloaded from NCBI (see below) (Tyanova et al., 2016; Sayers et al., 2022) and common laboratory contaminants. Statistical analysis was performed in Perseus version 2.0.7.0 (Tyanova and Cox, 2018). Proteins were recognized as being expressed when they had label-free quantification intensities in one out of three biological replicates. No proteins were retrieved from the control cultures of MAR_2009_72. Analysis was performed with a biomass grown on galactose. Proteins were considered being upregulated when the *p*-value and the difference were positive in the statistical *t*-test analysis (Supplementary Tables S8–S12).

Reference genomes were downloaded from NCBI: *Maribacter forsetii* NZ_JQLH01000001.1 (download 2022/10/06), *Maribacter* sp. MAR_2009_72 NZ_VISB01000001.1 (2022/09/27), *Maribacter* sp. Hel_I_7 NZ_JHZW01000001.1, NZ_JHZW01000002.1, and NZ_JHZW01000003.1 (2022/10/06), *Maribacter dokdonensis* MAR_2009_60 NZ_LT629754.1 (2022/10/06), and *Maribacter dokdonensis* MAR_2009_71 NZ_FNTB00000000.1 (2022/07/18).

### Bioinformatic analyses

Protein annotation was refined using several databases. CAZymes were considered to be identified if two out of three search algorithms in dbCAN3 were positive in the web interface search (Zheng et al., 2023). The conserved domain database (CDD) (Lu et al., 2019), the SulfAtlas web interface (Stam et al., 2022), InterPro (Paysan-Lafosse et al., 2022), PULDB (Terrapon et al., 2018), DeepTMHMM (Hallgren et al., 2022), SignalP (Teufel et al., 2022), BlastKOALA (Kanehisa et al., 2016), and UniProt (The UniProt, 2023) provided additional information.

Alignments for the SusC and GH16_3 protein trees were performed using MAFFT online in automode (see Supplementary material for details; Katoh et al., 2019). SusC proteins encoded in laminarin utilizing loci were retrieved from publications (Krüger et al., 2019). The GH16_3 sequences were taken from CAZy (Cantarel et al., 2009). The trees were calculated using the maximum likelihood method in Mega 11 (Supplementary material; Tamura et al., 2021a).

Proteins homolog to the laminarinase of *M. forsetii* (WP_051941695.1) were identified by BlastP using the NCBI web service (Altschul et al., 1990). Genomes were downloaded from NCBI, and the annotations of seven open reading frames (ORFs) upstream and seven ORFs downstream of the homologous GH16_3 genes were analyzed manually for the presence of polysaccharide utilization loci as defined by a SusC/D pair and at least one CAZymes within eight ORFs.

For the visualization of the data, the following programs and packages were used: R version 4.3.2 (R Core Team, 2023), ggplot2 (Wickham, 2016), gggenes (Wilkins, 2023), and Proksee (Grant et al., 2023).

### Enzyme activity tests and sugar quantifications

Cell pellets stored at −20°C were lyzed in 1 mL 50 mM 3-(N-morpholino) propanesulfonic acid (MOPS) by sonification on ice using a Sonoplus HD70 Bandelin MS73 (BANDELIN, Berlin, Deutschland) with a titanium sonotrode MS73 (BANDELIN) for 4 min at 50% power and 50% cycle (0.5 s on and 0.5 s off). The soluble fraction was separated by centrifugation for 15 min at 16,000 × *g* at 21°C. Enzymes were inactivated by pasteurization (80°C for 1 h). Protein concentration was determined by the Bradford protein assay using bovine serum albumin as standard (Bradford, 1976). Laminarinase assays were performed with 10–25 μg protein—of cell lysate, soluble fractions or resuspended membrane fractions—and 250 μg laminarin in 100 μL MOPS for 20 h at 21°C. After vacuum drying at 45°C for 1 h (Eppendorf Concentrator plus, Eppendorf, Hamburg, Germany), the sugars were labeled with 2 μL of 0.15 M 8-amino-1,3,6-naphthalenetrisulfonate (ANTS) in the presence of cyanoborohydride and analyzed on 25% v/v acrylamide gels following a protocol for fluorophore-assisted carbohydrate electrophoresis (FACE) (Becker et al., 2017). Separation was performed at 100 V for 30 min, followed by 200 V for 60 min. Gels were documented using a Bio-Rad GelDoc EZ Gel Imaging System (Cambridge Scientific, Watertown, United States).

The activity of β-glucosidase was detected with 1 mM 4-nitrophenyl-glucopyranoside in 50 mM potassium phosphate, pH 7.0, and the quantification of nitrophenol at 405 nm using an extinction coefficient of 7,500 M^−1^ cm^−1^.

Glucose concentrations were quantified with a glucose oxidase–peroxidase assay in 0.2 mL microtiter wells (Elabscience, Wuhan, China) using a spectrophotometer (SPECTROstar^®^ Nano, BMG LABTECH, Ortenberg, Germany). Total carbohydrate concentration was determined by the phenol–sulfuric acid method with glucose as standard, adapted for a microtiter plate (DuBois et al., 1956). Then, 25 μL of sample were mixed with 15 μL of 5% phenol and then acidified with 100 μL of concentrated sulfuric acid. The microtiter plate was incubated for 20 min at 30°C, and the absorbance was measured at 490 nm using a plate spectrophotometer.

The mass spectrometry proteomics data have been deposited to the ProteomeXchange Consortium via the PRIDE (Perez-Riverol et al., 2022) partner repository with the dataset identifier: *Maribacter forsetii* PXD049038 and 10.6019/PXD049038; *Maribacter* sp. MAR_2009_72 PXD049039 and 10.6019/PXD049039; *Maribacter dokdonensis* MAR_2009_60 PXD049040 and 10.6019/PXD049040; *Maribacter dokdonensis* MAR_2009_71 PXD049041 and 10.6019/PXD049041; and *Maribacter* sp. Hel_I_7 PXD049042 and 10.6019/PXD049042.

## Results

### Growth of *Maribacter forsetii* on laminarin

**Figure 1:**
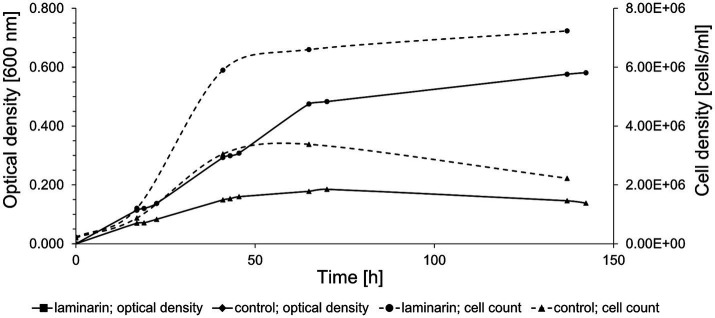
Growth curve of *Maribacter forsetii* in modified HaHa100V medium with 2 g/L laminarin at room temperature. The control culture has a limited amount of casamino acids. The optical density was measured at 600 nm, and the cell density was determined by cell counts using DAPI staining.

*Maribacter forsetii* KT02ds 18-6^T^ (DSM 18668^T^) grew with a maximal growth rate of μ = 0.09 h^−1^ at room temperature in a liquid mineral medium containing 0.3 g/L carbohydrate-free casamino acids as a limiting carbon source. The cell density reached an OD of 0.196. The addition of 2 g/L glucose or laminarin enabled growth rates of 0.10 and 0.09 h^−1^ and maximal OD of 0.578 and 0.501, respectively. The proliferation of cells was confirmed by counting DAPI-stained cells (Figure 1). Cell concentrations correlated with the optical density. Quantification of reactive aldehyde groups by the phenol-sulfuric acid method revealed a consumption of 42% for glucose and 62% for laminarin. Small amounts of reactive aldehydes (0.38 mM) were transiently detected during growth on casamino acids. These observations established an *in vivo* metabolism of laminarin by *Maribacter forsetii*.

### *Maribacter forsetii* utilizes specific proteins for laminarin degradation

**Figure 2:**
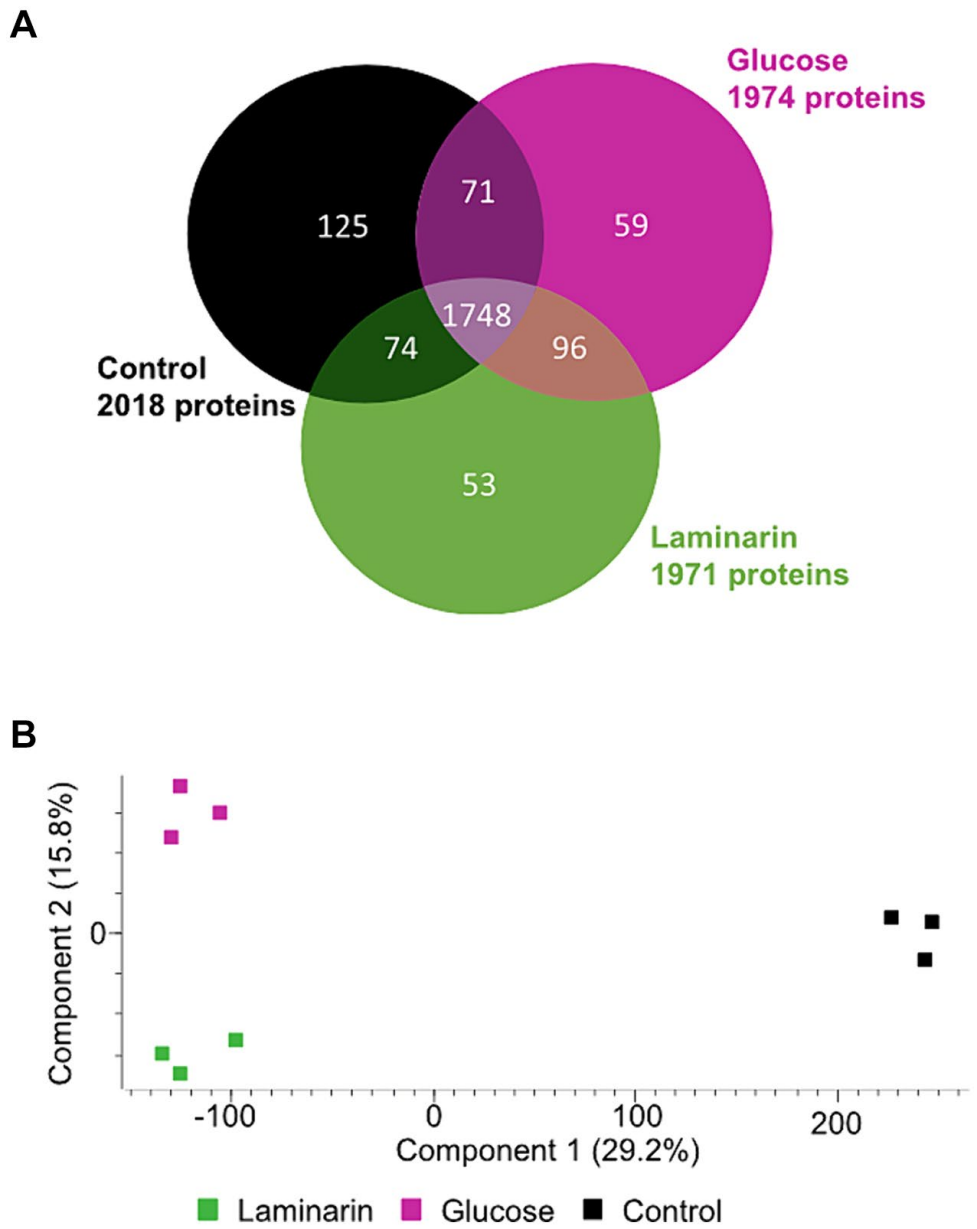
Comparison of three *Maribacter forsetii* proteomes of glucose, laminarin, and casamino acid grown cells. **(A)** Venn diagram showing the overlap of detected proteins in at least one of three biological replicates. **(B)** Principal component analysis showing the differences between the detected proteomes.

**Figure 3:**
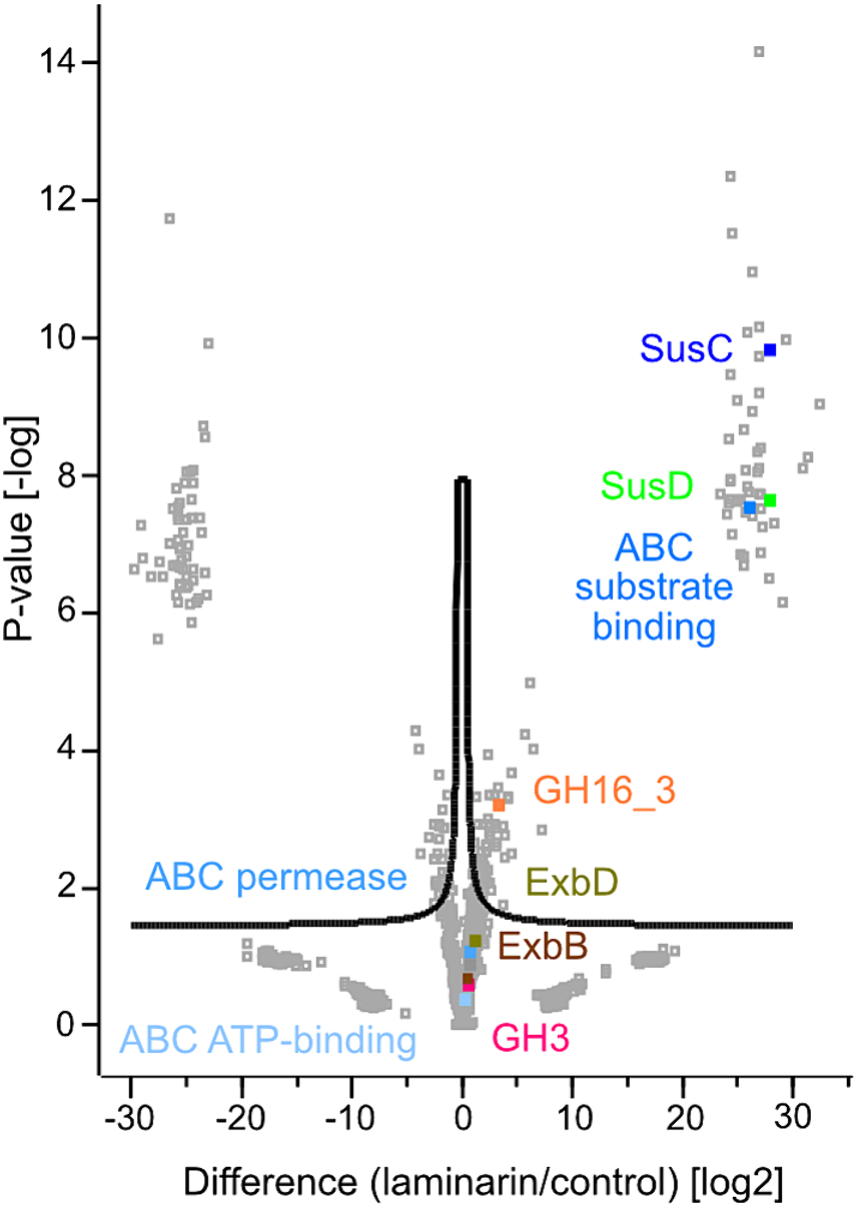
Volcano plot showing the statistical analysis of up- and downregulated proteins of laminarin and casamino acid grown *Maribacter forsetii* cells. Label free quantification (LFQ) intensities were transformed to log2. The mean value of the three biological replicates of either laminarin or control was used for the calculations. Missing observations were imputated in Perseus from NaN to constant value 0. FDR: 0.05, s0: 0.15.

The proteomes of the differently grown cultures showed significant variations. Overall, 1971 proteins were detected in laminarin-grown cells in at least one of the three biological replicates. We observed 1974 proteins in glucose-grown cells and 2018 proteins in cells from the control experiment grown with a limited amount of casamino acids. A total of 1748 proteins were detected at least once for each substrate. The analysis showed that 59 proteins were identified only in glucose-grown cells, 53 only in laminarin-grown cells, and 125 in the control culture cells (Figure 2A; Supplementary Table S3). A principal component analysis of the proteomes revealed a greater difference between the control and the sugar-derived proteomes and a smaller difference between the proteomes of glucose and of laminarin-grown cells (Figure 2B). Proteins of the basic cellular metabolism with an upregulation in the presence of laminarin included the enzymes involved in glycolysis, citric acid cycle, and oxidative phosphorylation as well as the pentose phosphate pathway. In addition, several proteins with homologies to characterized enzymes for the degradation of laminarin were upregulated (Figure 3; Table 1; Supplementary Table S8).

**Table 1:** Results of two sample *t*-test calculated for volcano plot visualization in Perseus (version 2.0.7.0) for enzymes highlighted in Figure 3, possible key role players in laminarin degradation in *Maribacter forsetii*.

Product	Difference	*p*-value	Significance	Protein id	Locus tag (NCBI)
GH16_3	3.20	3.20	+	WP_051941695.1	P177_RS20320
SusC	27.92	9.83	+	WP_245233002.1	P177_RS04595
SusD	27.91	7.65	+	WP_036152296.1	P177_RS04600
GH3	0.46	0.60		WP_157486485.1	P177_RS07335
ABC substrate-binding	26.00	7.55	+	WP_036150709.1	P177_RS00465
ABC permease	0.72	1.07		WP_036156362.1	P177_RS15985
ABC ATP-binding	0.26	0.38		WP_036150993.1	P177_RS01135
ExbD	1.05	1.25		WP_036151815.1	P177_RS03320
ExbB	0.49	0.67		WP_036151807.1	P177_RS03305

Difference indicates the difference between the label-free quantification values in log2 of the laminarin proteome versus the control proteome (mean values of the three biological replicates); *p*-value in −log10; significance: + sign shows if the specific lies above or below the significance (*p*-value) threshold. Missing observations were imputated in Perseus from NaN (Not a Number) to constant value 0. False discovery rate (FDR): 0.05, artificial within groups variance (default: 0). It controls the relative importance of the *t*-test p-value and the difference between means. At s0 = 0 only the p-value matters, while at non-zero s0 also the difference of means plays a role (s0: 0.15) (Tusher et al., 2001).

A glycosyl hydrolase family GH16_3, assigned to laminarinases as a β-glucan degrading endohydrolase (P177_RS20320), was upregulated in comparison with the control sample. The protein was predicted to be extracellular, as expected for the degradation of the polysaccharide. It was not detected in the proteome of glucose-grown cells. To support the GH16_3 characterization as an endo-β-1,3-laminarinase of *Maribacter forsetii*, we calculated a phylogenetic tree to examine the association with other GH16_3 proteins that have been characterized experimentally as either endo-β-1,3-laminarinase or endo-β-1,4-galactosidase (Supplementary Figure S1). The GH16_3 of *M. forsetii* was clustered together with GH16_3 from the other investigated *Maribacter* strains and with experimentally verified laminarinases. Separate branches included GH16_3 galactosidases as well as GH16_12 and GH16_16 of *Maribacter dokdonensis* MAR_2009_60 and MAR_2009_71.

The expected product of GH16_3 activity is a range of laminarin oligosaccharides which are actively transported into the periplasm by a TonB-dependent SusC/D transporter system (Labourel et al., 2015). Several SusC and SusD proteins were detected in the proteomes. The energy-delivering protein complex in the cytoplasmic membrane was expressed with two copies of ExbB (P177_RS03305 and P177_RS16115) and three copies of ExbD (P177_RS03320, P177_RS03315, and P177_RS16110) in the proteome. ExbBD proteins were upregulated in laminarin-grown cells (Supplementary Table S3). The SusC protein P177_RS04595 had the highest upregulation, followed by three other SusC proteins. The first one has a specific hit in the reverse position-specific iterated BLAST of NCBI-CDD, whereas the other three SusC proteins have only one superfamily hit, indicating that the annotation of the transporters is not clearly established. SusD (P177_RS04600) of the respective *susCD* gene pair was also highly upregulated. In addition, four more SusD proteins were upregulated. The aforementioned SusC/D pair was expressed in glucose-grown cells. These observations suggest the use of a SusC/D transport system in the laminarin metabolism.

The conversion of oligomeric laminarin to glucose in the periplasm can be catalyzed by a number of glycosyl hydrolases. Genome analysis revealed one GH1, two GH3, and two GH5 proteins; however, only one GH3 (P1177_RS07335) was expressed and upregulated in laminarin compared to glucose or control.

A candidate for the transport into the cell is an ABC transport system. The substrate-binding protein P177_RS00465 was highly expressed in laminarin- and glucose-grown cells. The permease P177_RS15985 and the ATP-binding protein P177_RS01135 may complete the ABC transporter. No major facilitator superfamily transporters were expressed in cells grown on laminarin, glucose, or casamino acids.

The phosphorylation of glucose to glucose-6-phosphate is a kinase reaction. We found several expressed and upregulated kinases in the genome. The most upregulated kinase was classified as a carbohydrate kinase (P177_RS13360). The pathway tool KEGG BlastKOALA suggested the polyphosphate glucose phosphotransferase P177_RS04875. This enzyme is only upregulated in comparison with the control.

**Figure 4:**
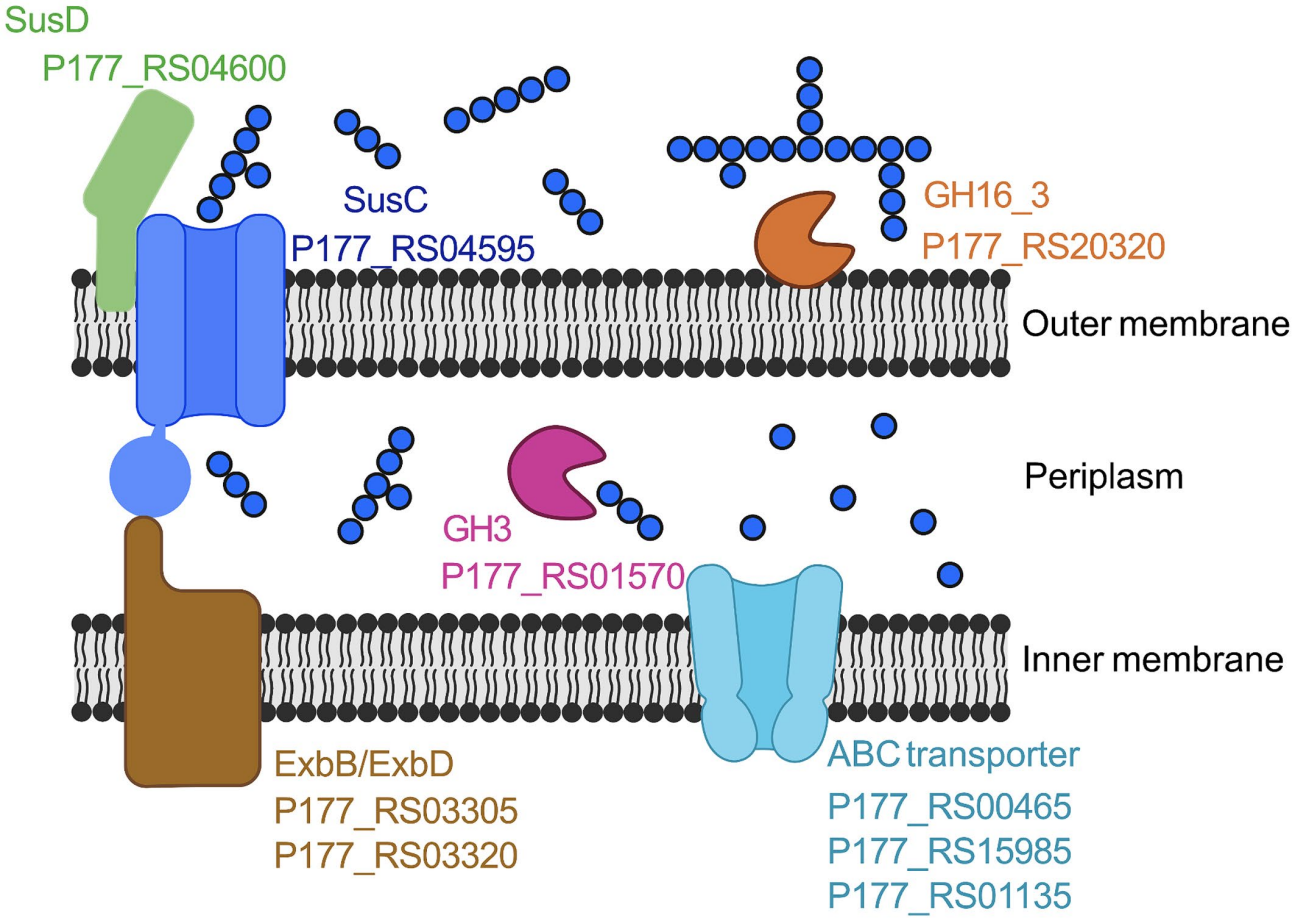
Proposed laminarin degradation pathway in *Maribacter forsetii*. GH16_3, laminarinase, P177_RS20320; SusD, nutrient uptake outer membrane protein (P177_RS04600); SusC, TonB-linked outer membrane protein (P177_RS04595); GH3, β-glucosidase (P177_RS01570); ExbB, proton channel family protein (P177_RS03305); ExbD, biopolymer transporter (P177_RS03320); ABC Transporter, ABC transporter substrate-binding (P177_RS00465), ABC transporter permease (P177RS_15985), and ABC transporter ATP-binding protein (P177_RS01135). Created with BioRender.com.

**Figure 5:**
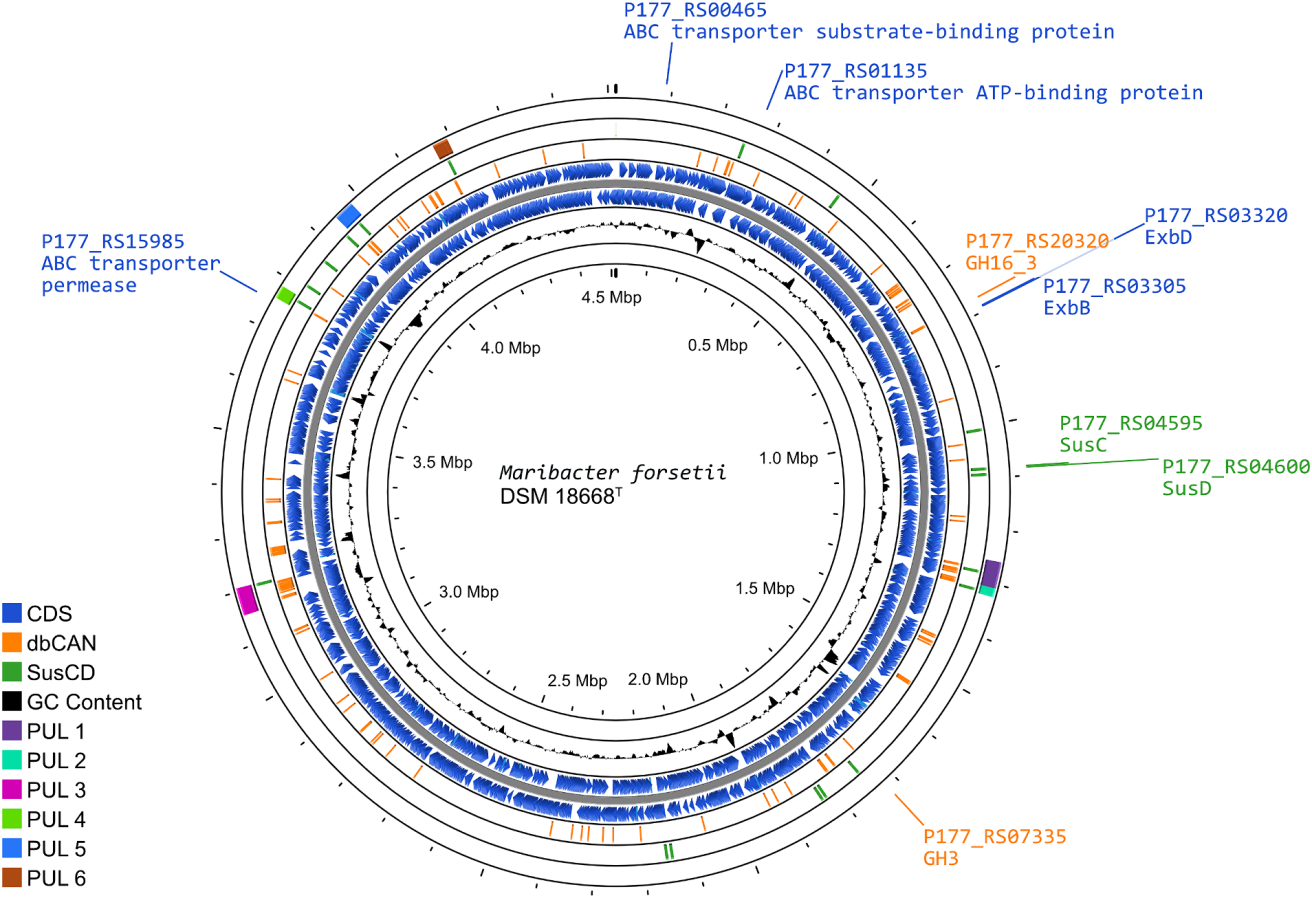
Representation of the *Maribacter forsetii* genome, displaying the overall genome structure, as well as the enzymes playing a possible key role in laminarin degradation. The rings (outside to inside), present (1) the location and length of polysaccharide utilization loci (PUL), defined by PULDB and Kappelmann et al. (2019) (purple); (2) the location of *susC* and *susD* genes, with SusCs having the motif Tigr04056 (green); (3) genes for CAZymes annotated by dbCAN3, annotated in at least two out of three search algorithms (orange); (4/5) coding sequences in forward and reverse direction from NCBI genome annotation of *Maribacter forsetii* (blue); (6) G/C content.

**Figure 6:**
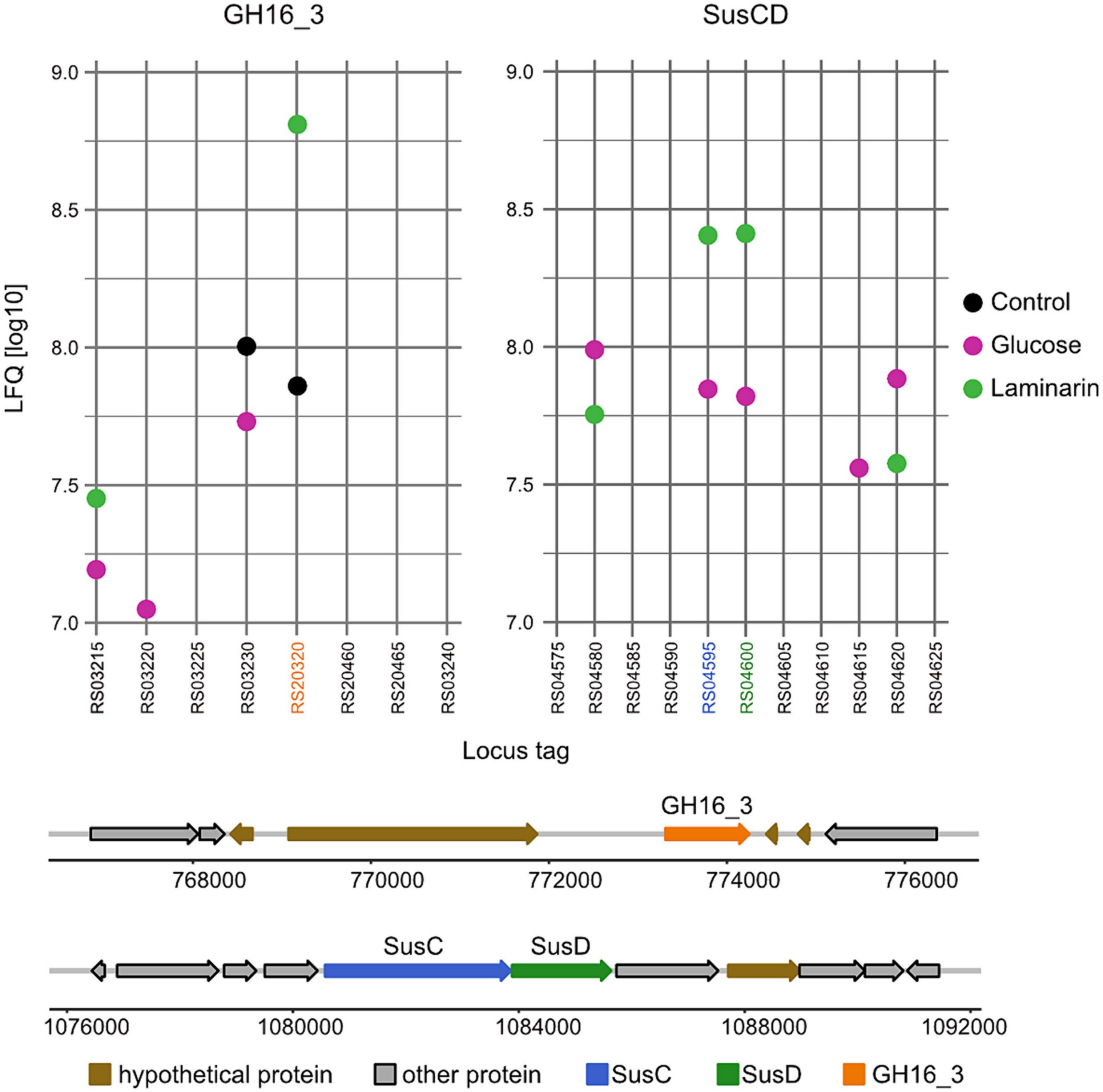
Gene organization and expression of enzymes proposed to degrade laminarin in *Maribacter forsetii*. Expression data are shown in LFQ values (log10) of the genetic region containing genes of GH16_3 and of the highest upregulated SusC/D pair of *Maribacter forsetii*.

These findings suggested a degradation pathway of laminarin by *Maribacter forsetii* similar to the pathways in free-living *Flavobacteriia* (Figure 4). The genes for these proteins are spread over the genome and not localized in a polysaccharide utilization locus (Figure 5). The neighboring genes of the GH16_3 gene (P177_RS20320, Figure 6) were not expressed in laminarin-grown cells. None of the SusC and SusD genes has a CAZyme in the neighborhood in the same reading direction. Other neighboring genes were expressed, in example for the SusC/D pair with the highest expression values in laminarin-grown cells (P177_RS04595 and P177_RS04600), alkaline phosphatase was expressed in laminarin and glucose, a FAD-dependent oxidoreductase in two out of three glucose samples and a phosphonatase-like hydrolase in two out three replicates in laminarin and glucose. The GH3 gene (P177_RS07335) has a neighboring anhydro-N-acetylmuramic acid kinase gene and more distantly a GH10 gene in the same reading direction. In coincidence with these findings, the genes for the ExbBD and ABC transporter proteins are widely distributed across the genome.

### Enzyme activities

**Figure 7:**
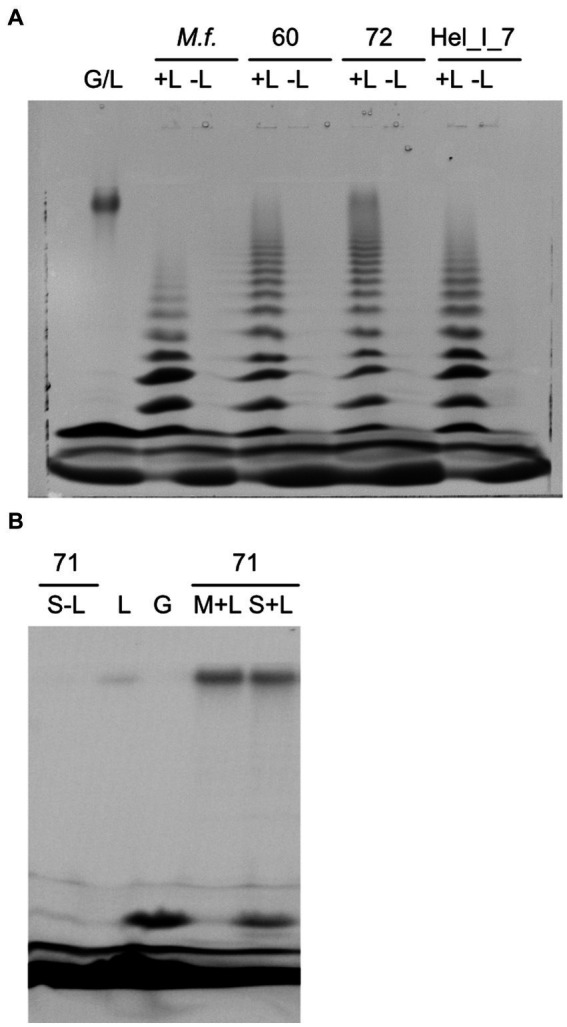
Hydrolysis of laminarin by soluble extracts of *Maribacter* visualized as fluorescently labeled sugars separated by polyacrylamide gel electrophoresis. **(A)** Strains with endo-laminarinase activity: G/L, standard of laminarin and glucose, +L/−L, assay with or without laminarin, *M.f*., *Maribacter forsetii*, 60, MAR_2009_60, 72, MAR_2009_72, Hel_I_7. **(B)** 71, MAR_2009_71 has no endo-laminarinase activity; S-L, assay of soluble fraction without laminarin; L, laminarin standard; G, glucose standard; M + L, assay of membrane fraction with laminarin; S + L, assay of soluble fraction with laminarin.

We investigated the presence of endo-laminarinase activity in cell extracts using the labeling of sugars with a fluorophore and size separation by acrylamide gel electrophoresis. Extracts of *Maribacter forsetii* and *Maribacter* sp. Hel_I_7, *Maribacter dokdonensis* MAR_2009_60, and *Maribacter* sp. MAR_2009_72 catalyzed the formation of a size range of oligosaccharides (Figure 7A), but the enzymes of *Maribacter dokdonensis* MAR_2009_71 did not show this laminarin hydrolysis (Figure 7B). The oligosaccharide formation is the characteristic pattern expected for an endo-laminarinase (Labourel et al., 2014, 2015). The activity was heat-labile, increased with increasing protein concentration, and was membrane-associated. It partly dissolved in MOPS buffer that had less ionic strength than seawater. Efficient removal of the enzyme activity from the membrane fraction was obtained by washing the membrane particles with detergents, including n-dodecyl-β-D-maltopyranoside, Tween 20, Tween 80, and Triton X-100 in buffers as described in Orwick-Rydmark et al. (2016). The endo-laminarinase activity of four strains confirmed the expression of the GH16_3 encoded in their respective genomes.

Extracts of *Maribacter dokdonensis* MAR_2009_71 showed a laminarin-dependent formation of a molecule with the retention time of glucose in FACE gels, catalyzed by the soluble fraction of the crude extract (Figure 7B). The formation of glucose by heat-sensitive enzymes was quantified by a glucose oxidase–peroxidase assay. Hydrolysis of 4-nitrophenyl-ß-D-glucopyranosid revealed a heat-labile β-glucosidase activity of 18 nmol s^−1^ (μg protein)^−1^ in soluble extracts. These observations indicated a different degradation pathway for laminarin in *Maribacter dokdonensis* MAR_2009_71. In addition, the absence of a GH16_3 gene in the genome coincided with the absence of an oligosaccharide ladder in the enzyme assay.

### Genomes and proteomes of other *Maribacter* strains

Homologues of the laminarin-degrading proteins of *Maribacter forsetii* were also detected in the other investigated *Maribacter* strains (Table 2; Supplementary Tables S4–S7). The endo-laminarinase activity coincided with the GH16_3 genes in the four genomes. The proteomic analyses revealed higher LFQ values for the corresponding proteins only for *Maribacter forsetii* and MAR_2009_72 grown on laminarin. It was not detected in the proteomes of Hel_I_7 and MAR_2009_60. This showed the sensitivity limitations of proteomics in this study in comparison with enzyme assays. No other genes for an endo-laminarinase were detected in the genomes. Other glycosyl hydrolase family GH16 genes were GH16_12 and GH16_16 in MAR_2009_60 and MAR_2009_71, annotated in the respective genomes as β-porphyranase and β-agarase (Viborg et al., 2019).

**Table 2:** Comparison of proteomic data sets of laminarin-grown cells of *Maribacter forsetii*, *Maribacter* sp. MAR_2009_72, *Maribacter dokdonensis* MAR_2009_60, *Maribacter dokdonensis* MAR_2009_71, and Maribacter sp. Hel_I_7; numbers of proteins expressed in laminarin-grown cells/annotated in the genome.

	*M. forsetii*	MAR_2009_72	MAR_2009_60	MAR_2009_71	Hel_I_7
GH16_3	1/1	1/1	0/1	0/0	0/1
GH16_12	0/0	0/0	0/1	0/1	0/0
GH16_16	0/0	0/0	0/1	0/1	0/0
SusC	10/19	10/22	7/23	5/24	6/16
SusC-specific hit	4/12	5/14	4/17	2/15	2/12
SusC-superfamily hit	6/7	5/8	3/6	3/9	4/4
SusD	12/20	8/22	7/23	9/24	6/21
GH1	0/1	0/3	0/1	0/1	0/1
GH3	1/4	2/6	0/4	0/5	0/5
GH5	0/2	0/2	0/1	0/2	0/2
GH17	0/0	0/0	0/0	0/0	0/0
GH30	0/0	0/0	0/0	0/0	0/0
ExbB	2/2	2/2	2/2	2/2	1/2
ExbD	3/5	3/5	3/3	3/3	2/5
ABC substrate-binding	2/4	1/1	0/3	0/3	0/2
ABC permease	2/19	1/17	0/20	0/21	0/21
ABC ATP-binding	5/17	8/16	1/19	1/18	2/19

The degradation of oligomeric laminarin can be performed by enzymes affiliating to GH1, GH3, and GH5, which were encoded in all five genomes, sometimes in multiple copies. Only MAR_2009_72 had expressed GH3 proteins, in this case two GH3 proteins. ABC transporters were encoded in all genomes. A complete ABC transport system was expressed in MAR_2009_72; in addition, *Maribacter forsetii* and ABC ATP-binding proteins were expressed in the other three strains. The specificity of these proteins is unknown. All five *Maribacter* strains had an expressed polyphosphate glucose phosphotransferase, classified as EC 2.7.1.63, most likely responsible for the conversion of glucose to glucose-6-phosphate after the transport into the cytoplasm.

The contrasting enzymatic activities of the two strains of *Maribacter dokdonensis* in our study have likely a molecular basis in the strain-specific part of the genomes. MAR_2009_60 has an endo-laminarinase GH16_3, and its degradation pathway appears to be similar to *Maribacter forsetii*. To characterize strain MAR_2009_71, we analyzed the proteomes and especially the strain-specific genes. The genome encodes 228 proteins that are absent in MAR_2009_60 and 406 proteins with less than 70% identity to the best Blast hit among the proteins in MAR_2009_60. The strain-specific genes are located in one large island of 206 genes and several smaller islands of 9 to 44 genes, in addition to individual genes. The genome encodes 60 glycosyl hydrolases, including 10 strain-specific ones. dbCAN predicted 47 glycosyl transferases, including 10 GT4 and 4 other GT with a strain specificity. One strain-specific GT4 (BLW30_RS12510) was expressed in laminarin-grown cells. The gene is part of a strain-specific genetic island of 26 genes (BLW30_RS12470—BLW30_RS12590) comprising 9 upregulated genes including a sugar transferase, a second GT4, and a nucleotide sugar dehydrogenase. A GT4 with 57% identity to the aforementioned enzyme was upregulated in MAR_2009_60.

In MAR_2009_71, the other upregulated GH or GT was a GH109 (BLW30_RS06230), whereas the homolog in MAR_2009_60 (BLT83_RS16160) was not upregulated on laminarin compared to the control. Proteomic analysis of MAR_2009_71 showed the upregulation of three SusC, four SusD, and a sugar phosphate isomerase/epimerase (Supplementary Table S6). A significant upregulation in both MAR_2009_71 and MAR_2009_60 was detected for three proteins annotated as SusC, an outer membrane protein, and a sensor protein.

**Figure 8:**
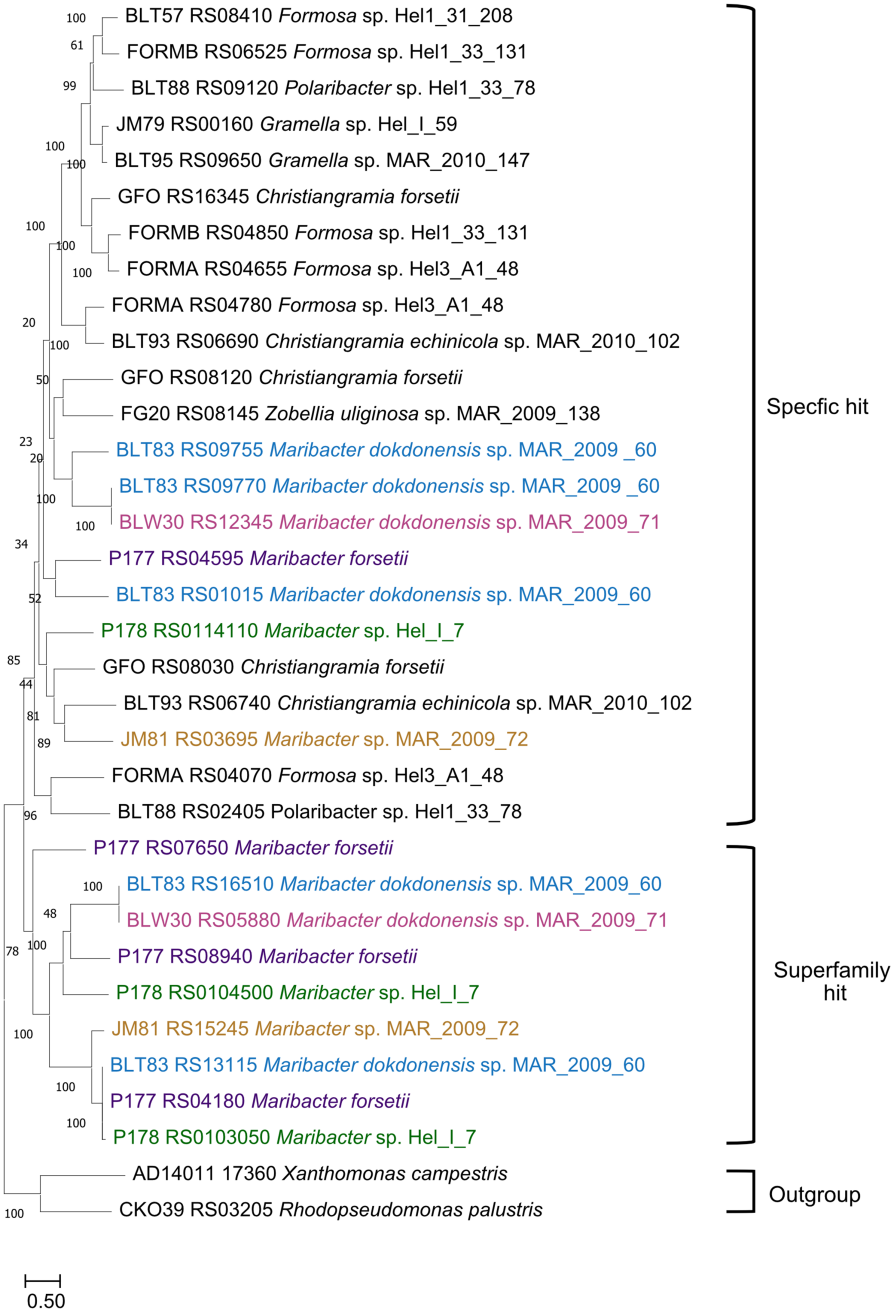
Phylogenetic tree showing the likeness between previous published SusCs affiliated with laminarin degradation and upregulated SusCs from the five *Maribacter* strains in this study. The evolutionary history was inferred by using the maximum likelihood method and Le_Gascuel_2008 model (LG + F + G4). The percentage of trees in which the associated taxa clustered together is shown next to the branches. Initial tree(s) for the heuristic search were obtained automatically by applying Neighbor-Join and BioNJ algorithms to a matrix of pairwise distances estimated using the JTT model and then selecting the topology with superior log likelihood value. A discrete Gamma distribution was used to model evolutionary rate differences among sites [4 categories (+*G*, parameter = 5.1185)]. The tree is drawn to scale, with branch lengths measured in the number of substitutions per site. Each SusC was analyzed using the CDD for identification of its specificity. The strain nomenclature was used as provided in NCBI (Feb.2024). *Maribacter forsetii* purple; *Maribacter* sp. Nov Hel_I_7 green; *Maribacter* sp. MAR_2009_72 orange; *Maribacter dokdonensis* MAR_2009_60 blue; *Maribacter dokdonensis* MAR_2009_71 pink.

All strains had expressed and upregulated SusC/D proteins together with expressed ExbBD. In earlier studies, the substrate specificity of SusC proteins was predicted largely by proteomic and phylogenetic studies. We calculated a protein tree to see whether the upregulated SusCs from the investigated *Maribacter* strains were affiliated with published SusC proteins that had been identified to be involved in laminarin degradation (Figure 8; Supplementary Table S2). SusC proteins encoded in laminarin-specific PULs from other *Flavobacteriia* formed together with *Maribacter* SusC proteins with a specific hit for the TIGR04056 SusC motif one branch in the tree. Separated were the SusC proteins with only a superfamily hit forming a second clade close to the out-group. *Maribacter dokdonensis* MAR_2009_60 has three upregulated SusC proteins with a specific hit, whereas the other strains have only one SusC for the transport of laminarin-derived oligosaccharides. The *Maribacter forsetii* SusC is related to one of *Maribacter dokdonensis* MAR_2009_60. The other two SusCs from MAR_2009_60 affiliate with the one of MAR_2009_71, forming a species-specific branch. A third branch is formed by MAR_2009_72 and Hel_I_7 together with proteins of *Christiangramia* (ex *Gramella*).

### Uptake of fluorescently labeled laminarin

**Figure 9:**
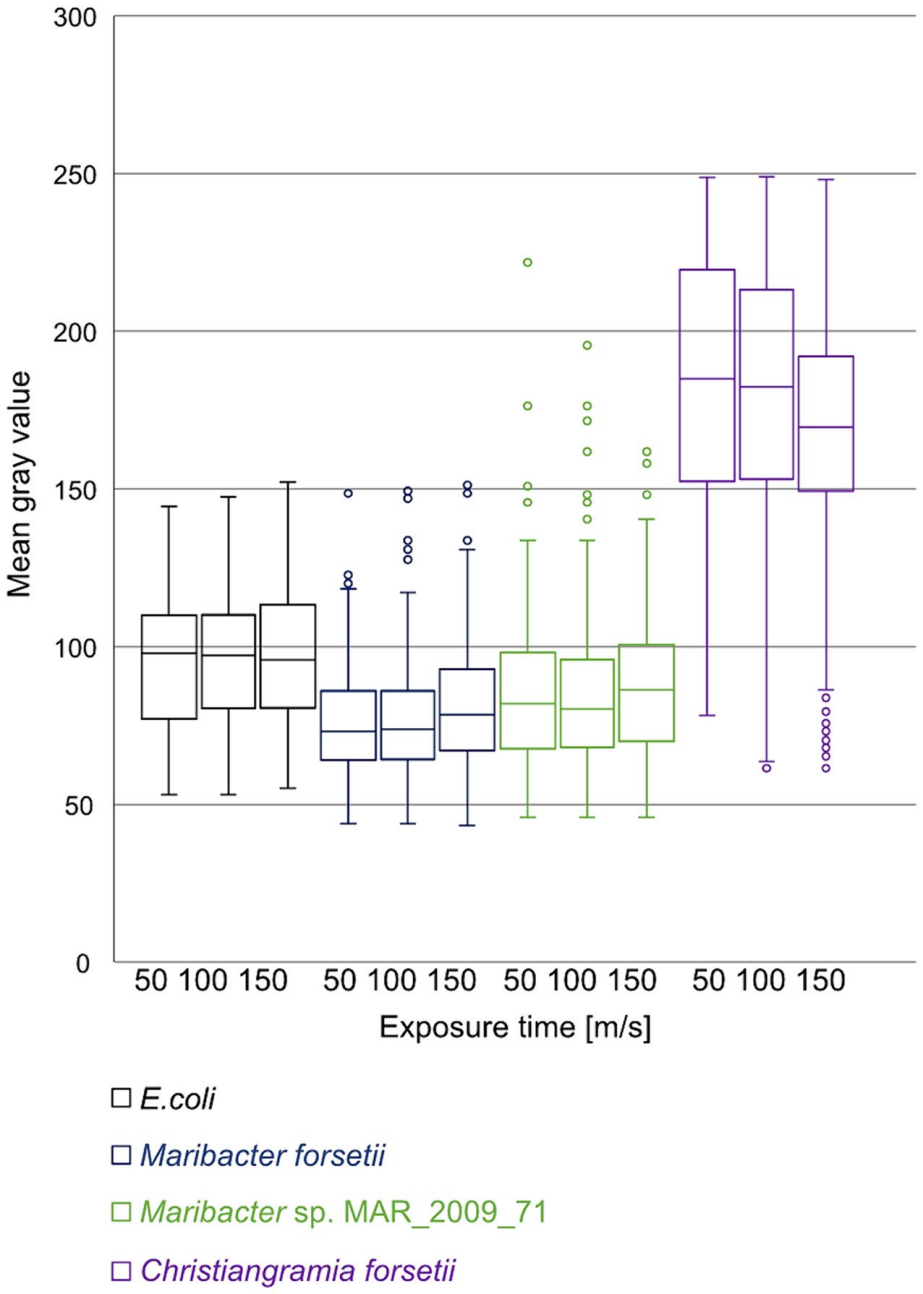
Mean gray value distribution of cells automatically detected as positive for uptake of labeled laminarin at 50, 100, and 150 ms exposure time. Images of cell samples after 1 min, 1, 2, 4, and 24 h were processed and automatically counted. Cell numbers were 68, 105, and 104 for *E. coli*, 261, 365, and 343 for *Maribacter forsetii*, 217, 308, and 332 for *Maribacter dokdonensis* MAR_2009_71, and 775, 2,459, and 1,635 for *Christiangramia forsetii* and represent areas of 10 filters identical in size.

The energy driven uptake of oligosaccharides via SusC/D proteins in the outer membrane is a key factor for the ecological success of *Flavobacteriia*. This transport has been demonstrated by the accumulation of fluorescently labeled sugars in the periplasm of free-living planktonic bacteria, referred to as selfish uptake (Reintjes et al., 2017; Fischer et al., 2019). None of the here investigated *Maribacter* strains showed selfish uptake following the standard protocol (Reintjes et al., 2017). To introduce a quantitative measure for this negative result, we switched from a threshold-integration method to a linear data acquisition and used the mean gray value (MGV) of the cells in the detection of the fluorescent signal (Figure 9). No specific uptake of laminarin was detectable for *Maribacter* strains and *E. coli DSM* 498 which served as negative control. In both cases, the automatic cell detection system identified a limited number of cells, approximately 10%, as positive. These cells had an MGV 10 units above the MGV of the background of the individual slide, in the overall range from 0 (black) to 255 (white). In contrast, *Christiangramia forsetii* cells showed a clear fluorescence signal for the selfish uptake by (i) an inducible process, (ii) a high cell number (~ 90% of DAPI cells), and (iii) a clear intensity 40 units above the background.

### *Maribacter forsetii* laminarinase homologues

The non-redundant protein dataset of NCBI included 57 homologous proteins that contained all domains of the *M. forsetii* laminarinase. Length varied between 687 and 705 AA. Lowest protein identity was 40%. *Maribacter* genomes encode 40 different homologues (Supplementary Table S13). The genes were not engulfed within PULs in all *Maribacter* genomes analyzed, including *M. algarum*, *M. aquivivus* DSM 16478, *M. arcticus* DSM 23546, *M. confluentis*, *M. huludaoensis*, *M. hydrothermalis*, *M. litoralis*, *M. sedimenticola* DSM 19840, *M. spongiicola*, *M. stanieri* DSM 19891, *M. zhoushanensis*, *M.* sp. 6B07, *M.* sp.1_MG-2023, *M.* sp. BPC-D8, *M.* sp. MJ134, and *M.* sp. 4 U21. Outside of *Maribacter*, homologues in *Aurantibacter* sp., *Eudoraea adriatica*, *E. chungangensis*, *Zobellia uliginosa*, *Z. barbeyronii*, *Croceitalea vernalis, and two* Flavobacteriaceae (ASW18X and TMED265) were not part of a PUL. The large gene was encoded within PULs in *Croceitalea dokdonensis*, *C.* sp. P059, *Croceivirga radicis*, and *Flavobacterium* ASW18X (86.3% ANI to *C. radicis*). In addition to a SusC/D pair, these PULs had either a catalytic GH16 domain protein or GH2, GH17, and several carbohydrate-binding module (CBM) proteins. *Croceitalea* sp. MZPC5 had a CAZyme cluster including a GH16 catalytic domain protein, GH17, two CBM proteins, and an MFS transporter.

## Discussion

Our study provided insight into the laminarin utilization in particle-associated *Maribacter* strains. They are capable of growing on laminarin as major carbon source. A previous *in silico* study did not detect laminarin PULs in *Maribacter* genomes (Kappelmann et al., 2019). Analyzing the proteomes, we detected the expression of enzymes known to be involved in laminarin degradation. Their genes have, however, a genetic neighborhood that did not fulfill definitions for canonical PULs: physically linked CAZyme genes located around a *susCD* pair whereby different search windows were applied (Grondin et al., 2017; Unfried et al., 2018; Krüger et al., 2019; Francis et al., 2021; Sidhu et al., 2023). Our study demonstrated that the absence of genetic loci should not be used to predict physiological traits. In our understanding, laminarin is not a major substrate for particle-associated *Maribacter*, and therefore, there was no selection for tight coregulation in one genetic locus.

The expressed GH16_3 is annotated as a laminarinase subfamily, a large and diverse subfamily, which can be found in all four kingdoms (Viborg et al., 2019). The GH16-laminarinase-like domain is the active site of hydrolysis. It has a size of 230 AA and is highly conserved; however, proteins with this domain are present in proteins up to over 1,700 AA in nearly 50 protein superfamilies, usually together with specific carbohydrate-binding domains or as yet unannotated regions, according to protein family models in CDD of NCBI. All these proteins are usually annotated in genomes as family 16 glycosyl hydrolase or glycoside hydrolase family 16 protein on the basis of the presence of the GH16-laminarinase-like domain.

The GH16_3 enzyme of *Maribacter forsetii* and other *Maribacter* strains is a large protein of ~700 AA and has homologues in some flavobacterial genomes. Our analysis resulted in the following annotation for the *Maribacter forsetii* GH16_3: a flexible N-terminal signal peptide for the sec secretion system (AA 1–20) with a lipid attachment site (AA 1–18), a polycistronic kidney disease (PKD) domain at AA 48–109, a laminarin-binding domain, and the catalytic site GH16-laminarin-like domain that is interrupted by a domain of unknown function. The seven-strand β-sandwich domain annotated as PKD domain can bind the protein onto the outer membrane because the characteristic feature of β-sandwiches is a hydrophobic site (Bycroft et al., 1999; Cheng et al., 2013). Laminarin binding by the whole N-terminus including the second domain (AA 6–294) is based on homology to the laminarin-binding surface glucan binding protein B of *Bacteroides fluxus* BfSGBP-B (Tamura et al., 2021b). In the *Bacteroides fluxus* PUL, the gene for BfSGBP-B is followed by a GH16_3 gene with a BACON domain (*Bacteroides*-associated carbohydrate-bind (putative) domain) preceding the GH16-laminarin-like domain (WP_034523369.1). In the *Maribacter* enzyme, the active hydrolase site is only preceded by the laminarin-binding domain, but the GH16 domain is interrupted after the β-sheet 5, following the nomenclature of *Zobellia* endo-laminarinase. LamA from *Zobellia galactivorans* has a small additional loop between the β-sheet 5 and the β-sheet 6, after AA 213 of LamA or AA 81 of LamC (Labourel et al., 2015). The inserted domain of unknown function in the *Maribacter* enzymes was not assigned by NCBI/CDD or InterPro to a described domain. It was with 29% identity over 290 AA related to the aforementioned laminarin-binding surface glucan binding protein B of *Bacteroides fluxus*, BfSGBP-B. The GH16-laminarin-like domain has the expected active site consisting of 67 amino acids and the WPA motif (Viborg et al., 2019). These homologies suggest that the large extracellular GH16_3 enzymes of *Maribacter* and other *Flavobacteriia* have two laminarin-binding domains in addition to the catalytic site with an endo-β-(1,3)-laminarinase activity. A model of the GH16_3 of *Maribacter* sp. MAR_2009_72 published by the European Bioinformatics Institute confirmed the domains.^2^

Recent studies have enlarged the number of enzyme activities associated with the GH16-laminarinase-like domain but also provided more insight into the substrate specificity of GH16_3 enzymes (Matard-Mann et al., 2017; Crouch et al., 2020). Determinants are the amino acids in the six fingers. A finger is a succession of β-strand + tight turn or loop or helix + β-strand extending from the protein “bowl” (Matard-Mann et al., 2017). A detailed comparison of GH16 glucanases, laminarinases, porphyranases, carrageenases, xyloglucanases, and other activities was the basis for a deeper bioinformatics analysis of *Maribacter* GH16_3 laminarinases (Crouch et al., 2020). Galactosidases have in finger one a conserved motif (WKLCTYN**N**A**W**SQ) with Asp and Trp blocking an equatorial C4 hydroxyl group of glucose (Crouch et al., 2020). This feature was not conserved in *Maribacter* GH16_3 laminarinases indicating sufficient space for glucose is available. This difference is also reflected in the phylogenetic GH16_3 analysis.

A feature conserved in β-(1,3)-glucanases, but not in GH16 enzymes with other activities, is two Trp’s before and after finger three. *Maribacter* GH16_3 laminarinases have these Trp’s within the motif GGTWPAL**W**ALGANFDEVG**W**P. Together with the endo-laminarinase-typical oligosaccharide degradation pattern—in contrast to strain MAR_2009_71, which has no GH16_3, uses an unidentified degradation system, and may be considered as negative control for the presence of endo-laminarinases—and the presence of laminarin-binding domains in the GH16_3 proteins, we conclude that the identified GH16_3 proteins are endo-laminarinases. Definitely, ultimative proof will be a future biochemical study on the importance of two laminarin-binding domains in the action of this laminarinase.

Uptake of laminarin oligo- and polysaccharides into the periplasm requires an outer membrane transport system. SusC/D transport systems for laminarin oligosaccharides were originally identified as expressed proteins in laminarin-grown biomass, a direct biochemical assay does not exist. All five *Maribacter* strains expressed at least one SusC protein that has a specific similarity to the TIGR04056 motif for sugar-transporting SusC proteins. The motif includes the N-terminal extension of TonB-dependent transporter (PFAM13715) characteristic for SusC (Pollet et al., 2021). Our phylogenetic analyses affiliated the expressed SusC proteins with laminarin-specific SusC’s of other *Flavobacteriia*. The gene *susC* of *Maribacter forsetii* (P177_RS4595) did not encode an N-terminal signal peptide domain. This detail was also observed in the genomes of *Formosa* strain A and B and *Christiangramia forsetii* (FORMA_RS04655, FORMB_RS04850, and GFO_RS08120).

For oligosaccharide hydrolysis in the periplasm, β-exo-glucanases of the GH3 family were expressed. The *Maribacter forsetii* gene P177_RS07335 is predicted to have the periplasmic β-D-glucosidase domain (COG1472) and a C-terminal uncharacterized 140 AA region. A constraint alignment with the larger C-terminal domain of barley GH3 isozyme ExoI indicated conserved amino acids, suggesting a glucan binding for the domain (Varghese et al., 1999). Transport of glucose into the cytosol is presumably performed via an ABC transport system. Two complete systems consisting of substrate-binding, permease, and ATP-binding protein were expressed in *Maribacter forsetii* when grown on laminarin. The pathway is expected to import glucose. The imported glucose can be further hydrolyzed in the glycolysis and then replenish the citric acid cycle.

Comparison of the laminarin utilization of *Maribacter forsetii* with the other four *Maribacter* strains of this study brought similarities but also differences to light. *Maribacter* sp. MAR_2009_72 uses the same degradation pathway for laminarin as *Maribacter forsetii*. All key enzymes were expressed in its proteome: GH16_3, SusC/D, ExbBD, GH3, and ABC transport system. The presence of GH16_3 in the genomes of MAR_2009_60 and Hel_I_7 and the endo-laminarinase activity of the cellular extracts suggested a similar pathway; however, our proteomic analysis did not detect the corresponding GH16_3 proteins. For *Maribacter dokdonensis* MAR_2009_71, a GH16_3 was not annotated in the genome and we observed the lack of endo-laminarinase activity. These findings suggested that growth of MAR_2009_71 on laminarin is based on a so-far-uncharacterized set of enzymes.

Free-living and particle-associated bacteria differ in their genome size and numbers of genes for polysaccharides degradation. For example, the genus *Winogradskyella* includes bacteria of both lifestyles, and the genome size correlates with the lifestyle, being that particle-associated bacteria have larger genomes (Alejandre-Colomo et al., 2021). These comprise more degradative functions and motility, either swimming or gliding. Within the particle-associated bacteria, many particle-attached bacteria have the capacity to glide on surfaces (Seymour et al., 2017). All five *Maribacter* strains have the machinery for gliding annotated in their genome. We found most gliding-associated enzymes expressed in the proteomes, and gliding was observed on plates. The investigated *Maribacter* strains have a range of PULs, including ones for alginate and alpha-glucans (Kappelmann et al., 2019). The separation into free-living and particle-associated bacteria does not coincide with the genus border. *Winogradskyella* and *Polaribacter* include strains with both lifestyles and PUL for laminarin (Xing et al., 2015; Avcı et al., 2020; Alejandre-Colomo et al., 2021). *Christiangramia* (ex *Gramella*) is particle-associated and has a PUL for laminarin (Kabisch et al., 2014).

This leaves the question open why has a laminarin utilization loci not evolved in *Maribacter*? Settlement experiments showed a diatom-associated lifestyle of *Maribacter* (Heins et al., 2021b). We argue that laminarin is rarely available for *Maribacter* in relation to algal surface polysaccharides and algal exudates. Laminarin is an intracellular carbon storage for algae and concealed for bacteria. Only upon algal lysis, an ephemeral event, laminarin becomes available, but diffuses rapidly away, with a diffusion coefficient of one-third of that of glucose (Elyakova et al., 1994). Thus, laminarin appears to have little effect on how the genomes of particle-associated *Maribacter* strains continues to evolve. We interpret this absence of a laminarin PUL as evidence for a specialization on less soluble algal polysaccharides and algal exudates.

Bacterioplankton ecotypes are often defined by experimental methods, that is, the separation into free-living and particle-associated bacteria by sequential filtration. Alternative separation method, in example settlement, gave access to free-living motile bacteria of the phycosphere and separated large free-living bacteria from bacteria attached to settling particles. Living alga with a constant production of exudates differ from marine snow particle that represent a single-fed batch substrate. According to our substrate flux hypothesis, living on laminarin-rich dead organic matter may support the evolution of laminarin PULs in particle-associated bacteria specialized on dead particles. Homologs of the laminarinase of *M. forsetii* are present in laminarin PULs together with GH17, GH2, and other CAZymes in *Croceivirga radicis*, isolated from a rotten mangrove root, *Croceitalea dokdonensis*, isolated from the rhizosphere of the brown alga *Ecklonia kurome*, and two *Croceitalea* strains from sea surfaces. The presence of the large laminarinase gene outside and within PULs reflects the huge diversity of niches for microbial life in nature.

## Data availability statement

The datasets presented in this study can be found in online repositories. The names of the repository/repositories and accession number(s) can be found in the article/Supplementary material.

## Author contributions

SK: Writing – review & editing, Writing – original draft, Visualization, Methodology, Investigation, Formal analysis, Data curation, Conceptualization. DZ: Methodology, Writing – review & editing, Supervision. GR: Writing – review & editing, Methodology, Investigation, Funding acquisition, Formal analysis. KR: Writing – review & editing, Resources, Funding acquisition. RA: Writing – review & editing, Supervision, Resources, Funding acquisition. JH: Writing – review & editing, Writing – original draft, Validation, Supervision, Project administration, Methodology, Investigation, Funding acquisition, Conceptualization.

## Footnotes

^1^ https://www.mpi-bremen.de/en/automated-microscopy.html#section19794

^2^ https://alphafold.ebi.ac.uk/entry/A0A559NTH3
