# Supplementary material for: Genes for laminarin degradation are dispersed in the genomes of particle-associated Maribacter species
Source: Front Microbiol. 2024 Aug 12;15:1393588. doi: 10.3389/fmicb.2024.1393588 (PMC11345257; doi:10.3389/fmicb.2024.1393588)
Supplement: Supplementary file 1 [file Data_Sheet_1.DOCX]

**Genes for laminarin degradation are dispersed in the genomes of particle-associated *Maribacter* species**

Saskia Kalenborn^1^, Daniela Zühlke^2^, Greta Reintjes^3^, Katharina Riedel^2^, Rudolf I. Amann^1^, Jens Harder^1*^

^1^Department of Molecular Ecology, Max Planck Institute for Marine Microbiology, Bremen, Germany; ^2^Department for Microbial Physiology and Molecular Biology, University of Greifswald, Germany; ^3^ Microbial Carbohydrate Interaction Group, Department of Biology and Chemistry, University of Bremen, Germany

Corresponding author mail: [jharder@mpi-bremen.de](mailto:jharder@mpi-bremen.de)

Supplement

Supplement Figure 1


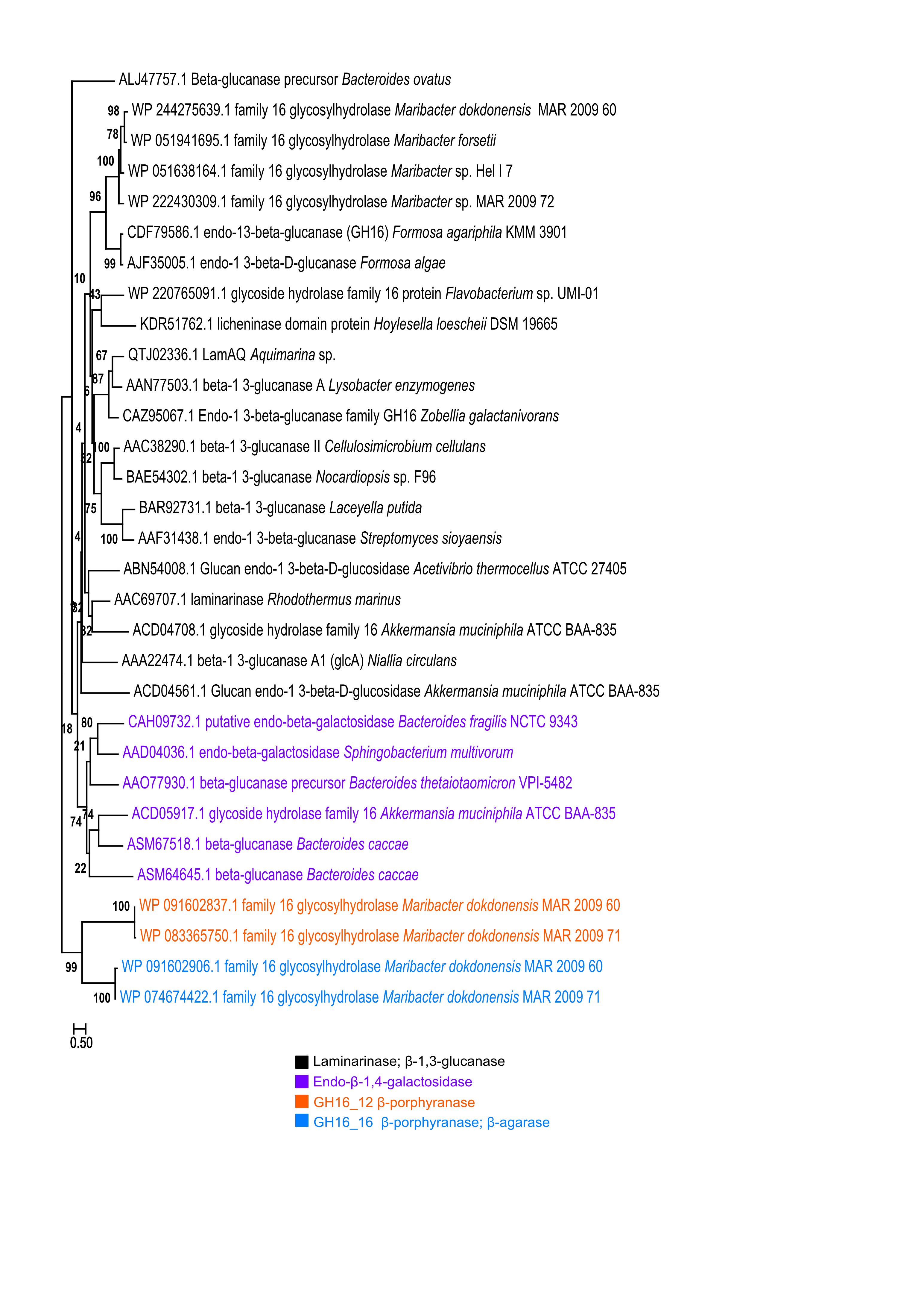


Supplement figure 1: Phylogenetic tree shows the likeness between enzymes of the GH16_3 family, characterised in the CAZy database and the ones from the five Maribacter strains in this study. The evolutionary history was inferred by using the Maximum Likelihood method and Le_Gascuel_2008 model (LG+F+G4). The percentage of trees in which the associated taxa clustered together is shown next to the branches. Initial tree(s) for the heuristic search were obtained automatically by applying Neighbor-Join and BioNJ algorithms to a matrix of pairwise distances estimated using the JTT model, and then selecting the topology with superior log likelihood value. A discrete Gamma distribution was used to model evolutionary rate differences among sites (4 categories (+*G*, parameter = 3.7828)). The tree is drawn to scale, with branch lengths measured in the number of substitutions per site.

Supplement Methods

Parameter for MaxQuant analysis as provided by the software

Parameter Value

Version 2.2.0.0

Include contaminants True

PSM FDR 0.01

PSM FDR Crosslink 0.01

Protein FDR 0.01

Site FDR 0.01

Use Normalized Ratios For Occupancy True

Min. peptide Length 7

Min. score for unmodified peptides 0

Min. score for modified peptides 40

Min. delta score for unmodified peptides 0

Min. delta score for modified peptides 6

Min. unique peptides 0

Min. razor peptides 1

Min. peptides 1

Use only unmodified peptides and True

Modifications included in protein quantification Oxidation (M);Acetyl (Protein N-term)

Peptides used for protein quantification Razor

Discard unmodified counterpart peptides True

Label min. ratio count 2

Use delta score False

iBAQ True

iBAQ log fit True

Match between runs False

Find dependent peptides False

Decoy mode revert

Include contaminants True

Advanced ratios True

Fixed andromeda index folder

Combined folder location

Second peptides True

Stabilize large LFQ ratios True

Separate LFQ in parameter groups False

Require MS/MS for LFQ comparisons True

Calculate peak properties False

Main search max. combinations 200

Advanced site intensities True

Write msScans table False

Write msmsScans table True

Write ms3Scans table True

Write allPeptides table True

Write mzRange table True

Write DIA fragments table False

Write DIA fragments quant table False

Write pasefMsmsScans table True

Write accumulatedMsmsScans table True

Max. peptide mass [Da] 4600

Min. peptide length for unspecific search 8

Max. peptide length for unspecific search 25

Razor protein FDR True

Disable MD5 False

Max mods in site table 3

Match unidentified features False

Epsilon score for mutations

Evaluate variant peptides separately True

Variation mode None

MS/MS tol. (FTMS) 20 ppm

Top MS/MS peaks per Da interval. (FTMS) 12

Da interval. (FTMS) 100

MS/MS deisotoping (FTMS) True

MS/MS deisotoping tolerance (FTMS) 7

MS/MS deisotoping tolerance unit (FTMS) ppm

MS/MS higher charges (FTMS) True

MS/MS water loss (FTMS) True

MS/MS water loss (FTMS for cross link) False

MS/MS ammonia loss (FTMS) True

MS/MS ammonia loss (FTMS for cross link) False

MS/MS dependent losses (FTMS) True

MS/MS recalibration (FTMS) False

MS/MS tol. (ITMS) 0.5 Da

Top MS/MS peaks per Da interval. (ITMS) 8

Da interval. (ITMS) 100

MS/MS deisotoping (ITMS) False

MS/MS deisotoping tolerance (ITMS) 0.15

MS/MS deisotoping tolerance unit (ITMS) Da

MS/MS higher charges (ITMS) True

MS/MS water loss (ITMS) True

MS/MS water loss (ITMS for cross link) False

MS/MS ammonia loss (ITMS) True

MS/MS ammonia loss (ITMS for cross link) False

MS/MS dependent losses (ITMS) True

MS/MS recalibration (ITMS) False

MS/MS tol. (TOF) 25 ppm

Top MS/MS peaks per Da interval. (TOF) 16

Da interval. (TOF) 100

MS/MS deisotoping (TOF) True

MS/MS deisotoping tolerance (TOF) 0.01

MS/MS deisotoping tolerance unit (TOF) Da

MS/MS higher charges (TOF) True

MS/MS water loss (TOF) True

MS/MS water loss (TOF for cross link) False

MS/MS ammonia loss (TOF) True

MS/MS ammonia loss (TOF for cross link) False

MS/MS dependent losses (TOF) True

MS/MS recalibration (TOF) False

MS/MS tol. (Unknown) 20 ppm

Top MS/MS peaks per Da interval. (Unknown) 12

Da interval. (Unknown) 100

MS/MS deisotoping (Unknown) True

MS/MS deisotoping tolerance (Unknown) 7

MS/MS deisotoping tolerance unit (Unknown) ppm

MS/MS higher charges (Unknown) True

MS/MS water loss (Unknown) True

MS/MS water loss (Unknown for cross link) False

MS/MS ammonia loss (Unknown) True

MS/MS ammonia loss (Unknown for cross link) False

MS/MS dependent losses (Unknown) True

MS/MS recalibration (Unknown) False

Site tables Oxidation (M)Sites.txt

Parameters in Perseus 2.0.7.0

Volcanoplot

t-test

Side: both

Number of randomizations: 250

Preserver grouping in randomizations: None

FDR: 0.05

s0: 0.15

Principle component analysis

Number of components: 5

Cut-off method: Benjamini-Hochberg

Benjamini-Hochberg FDR: 0.05

Relative enrichment: None

Parameters for the phylogenetic trees

We used amino acid sequences of the visualized proteins.

Mafft alignment parameters in automode:

Scoring matrix for amino acid sequences: BLOSUM62

Gap opening penaltiy:1.53

Strategy: Auto (FFT-NS-1,FFT-NS-2, FFT-NS-i or L-INS-I; depends on data size)

Mega11:

The evolutionary history was inferred by using the Maximum Likelihood method and Le_Gascuel_2008 model (LG+F+G4), which was the best model in the model test based on BIC. The percentage of trees in which the associated taxa clustered together is shown next to the branches. Initial tree(s) for the heuristic search were obtained automatically by applying Neighbor-Join and BioNJ algorithms to a matrix of pairwise distances estimated using the JTT model, and then selecting the topology with superior log likelihood value. A discrete Gamma distribution was used to model evolutionary rate differences among sites (4 categories). The trees are drawn to scale, with branch lengths measured in the number of substitutions per site.
